# Randomized, controlled, two-arm, interventional, multicenter study on risk-adapted damage control orthopedic surgery of femur shaft fractures in multiple-trauma patients

**DOI:** 10.1186/s13063-016-1162-2

**Published:** 2016-01-25

**Authors:** Dieter Rixen, Eva Steinhausen, Stefan Sauerland, Rolf Lefering, Marc G. Maegele, Bertil Bouillon, Guido Grass, Edmund A. M. Neugebauer, and members of the Damage Control Study Group

**Affiliations:** Department of Orthopedic and Trauma Surgery, Berufsgenossenschaftliche Unfallklinik Duisburg, Großenbaumer Allee 250, 47249 Duisburg, Germany; Witten-Herdecke University, Faculty of Health, Witten, Germany; Institute for Research in Operative Medicine, University of Witten-Herdecke, Ostmerheimer Str. 200, 51109 Cologne, Germany; Department of Trauma and Orthopedic Surgery, University of Witten-Herdecke at the Hospital Cologne-Merheim, Ostmerheimer Str. 200, 51109 Cologne, Germany; Office of the Ethics Committee, Medical Faculty of the University of Cologne, Cologne, Germany

**Keywords:** Multiple-trauma, Damage control, Randomized study, SOFA score, Femur fracture

## Abstract

**Background:**

Long bone fractures, particularly of the femur, are common in multiple-trauma patients, but their optimal management has not yet been determined. Although a trend exists toward the concept of “damage control orthopedics” (DCO), current literature is inconclusive. Thus, a need exists for a more specific controlled clinical study. The primary objective of this study was to clarify whether a risk-adapted procedure for treating femoral fractures, as opposed to an early definitive treatment strategy, leads to an improved outcome (morbidity and mortality).

**Methods/Design:**

The study was designed as a randomized controlled multicenter study. Multiple-trauma patients with femur shaft fractures and a calculated probability of death of 20 to 60 % were randomized to either temporary fracture fixation with external fixation and defined secondary definitive treatment (DCO) or primary reamed nailing (early total care). The primary objective was to reduce the extent of organ failure as measured by the maximum sepsis-related organ failure assessment (SOFA) score.

**Results:**

Thirty-four patients were randomized to two groups of 17 patients each. Both groups were comparable regarding sex, age, injury severity score, Glasgow Coma Scale, prothrombin time, base excess, calculated probability of death, and other physiologic variables. The maximum SOFA score was comparable (nonsignificant) between the groups. Regarding the secondary endpoints, the patients with external fixation required a significantly longer ventilation period (p = 0.049) and stayed on the intensive care significantly longer (p = 0.037), whereas the in-hospital length of stay was balanced for both groups. Unfortunately, the study had to be terminated prior to reaching the anticipated sample size because of unexpected low patient recruitment.

**Conclusions:**

Thus, the results of this randomized study reflect the ambivalence in the literature. No advantage of the damage control concept could be detected in the treatment of femur fractures in multiple-trauma patients. The necessity for scientific evaluation of this clinically relevant question remains.

**Trial registration:**

Current Controlled Trials ISRCTN10321620

Date assigned: 9 February 2007.

**Electronic supplementary material:**

The online version of this article (doi:10.1186/s13063-016-1162-2) contains supplementary material, which is available to authorized users.

## Background

Trauma is a major medical and economical issue of healthcare systems today and the leading cause of death between the age of 1 and 45 years [1]. Although long-bone fractures, and particularly femur fractures, are common and often troublesome in multiple-trauma patients, the optimal fracture management in these patients is not yet resolved [2–4]. Thus, the question remains whether primary internal (nail/plate) or external fixation (fixateur externe) is advantageous for this patient population, especially in high-risk patients with additional chest or head injuries [2–4].

While nailing is considered the gold standard for treatment of isolated femur shaft fractures, it is compromised by the significant distress caused by operation time, blood loss, and insertion of the nail, which may act as a “second hit.” Studies comparing reamed and unreamed intramedullary nailing show the superiority of the reamed nail [5, 6]. On the other hand, advocates of temporary external fixation in multiple trauma patients assert its simplicity with regard to initial treatment, as well as hypothetical advantages regarding patient security with less blood loss and a reduction in the systemic response. However, possible disadvantages of temporary external fixation must also be considered (for example, planned additional surgery for the secondary definitive procedure or increased infection rates by conversion of external to internal fixation). Moreover the planned conversion within the first days after trauma may also act as a “second hit” to the patient, because the optimal time for conversion from external fixation to a definitive procedure is not clear [7, 8].

With respect to the question of “early total care” or temporary fracture fixation by external fixation in multiple-trauma patients, the literature presents a diversity of studies supporting different views. Neither evidence-based guidelines [2, 3] nor a systematic review [4] could clarify the optimal time point or the procedure of femoral fracture fixation in multiple-trauma patients. In addition, an analysis of the trauma registry of the German Trauma Society, which included more than 8,000 multiple trauma patients, showed that management differs widely and depends on the individual hospital strategy, as well as the patient characteristics [4].

In this respect, increasing literature evidence suggests that neither “early total care” nor temporary external fixation with secondary definitive internal osteosynthesis should be considered as standard therapy in *all* patients. Instead, decision making should be dependent on the patient’s individual risk according to the anatomic and physiologic injury severity (risk-adapted damage control concept). Unfortunately, to date, no proof exists for the superiority of the risk-adapted damage control concept based on conclusive randomized controlled clinical trials. Thus, a well-designed randomized study was urgently needed to clarify this question.

This study investigates whether the use of damage control through the application of external fixation to the femoral shaft fractures in severely injured multiple-trauma patients will reduce the risk of mortality as measured by the sepsis-related organ failure assessment (SOFA) score [9, 10] when compared to early intramedullary nailing.

## Methods/Design

This study was registered prospectively in a publicly accessible registry (Current Controlled Trials ISRCTN10321620). It was designed as a randomized, controlled, two-arm, interventional, multicenter study [11].

The inclusion criteria were multiple trauma (injury of at least two body regions) with an injury severity score (ISS) ≥ 16, a femoral shaft fracture which can be treated in principle by nail or fixateur externe (surgical treatment beginning within 24 hours after trauma), age ≥ 18 years, and a calculated probability of death between 20 % and 60 % [12–14].

Considering probability of death at randomization allowed an equal distribution of global prognosis in both treatment arms. The calculation of prognosis was performed with a validated method of estimating the probability of death in multiple trauma patients [12–14] using clinical data (age, ISS, Glasgow Coma Scale (GCS), prothrombin time and base excess (BE)). For better understanding, in Germany (and thus also in the trauma registry of the German Trauma Society), the prothrombin time is preferentially reported and documented as Quick’s value in percentage (100 % = normal). A Quick’s value of < 60 % is equivalent to a prothrombin time ratio of approximately 1.4 [15].

The exclusion criteria were III° open fractures, refusal of one of both strategies by either the investigator or the patient, start of internal or external fracture fixation before randomization, participation in concurrent interventional studies, or pregnancy.

Temporary fracture fixation with external fixation and secondary reamed intramedullary nailing was the experimental intervention. Secondary surgery could be performed as soon as the patients treated with external fixation were stabilized with ventilation (paO_2_/FiO_2_ > 200 if ventilated or no need for ventilation), coagulation (prothrombin time > 60 % and platelets > 60,000/μl), hemodynamics (no need for noradrenalin or adrenalin and mean arterial pressure > 60 mmHg), the metabolic system (BE > -6.0 mmol/l), and furthermore showed no signs of systemic or local inflammation. The control intervention, however, was primary reamed nailing of the femoral shaft fracture.

All multiple-trauma patients who presented to the participating hospitals with femur shaft fractures and age ≥ 18 years were recorded, and eligibility was checked (screening). The probability of death was calculated on the study website [12–14]. If all inclusion criteria were fulfilled, the patient was randomized and documentation began. Reasons for noninclusions were recorded. Allocation concealment was granted by internet randomization, whereby the type of surgery was given only after inclusion of the patient.

The primary endpoint was the reduction of organ failure as measured by the maximum SOFA score within 28 days after trauma. For the present study, the five organ SOFA score (excluding central nervous system) was used. Thus the maximum SOFA score was 20 points (4 points for each organ) [16]. The SOFA score was assessed daily for the first 28 days after trauma. Documentation began in the ICU and continued until the patient returned to the normal ward, where the SOFA score was set to zero. If the patient was discharged home within the first 28 days, the SOFA score was set to zero by definition. If the patient was transferred to another hospital, the last observation was continued until day 28. Patients who died during the first 28 days after trauma were assigned the maximum possible SOFA score (20 points) for each day after death.

Secondary endpoints were hospital mortality, cumulative organ failure (= sum of SOFA score points for the first 28 days), incidence of Acute Respiratory Distress Syndrome (ARDS) [17], incidence of Systemic Inflammatory Response Syndrome (SIRS) and sepsis [18] during intensive care unit (ICU) stay, length of ICU stay, as well as the number of days on ventilation, and the in-hospital length of stay.

The primary hypothesis was that the damage control principle is able to reduce the maximum SOFA score by 1 to 2 points. Data from Ferreira et al. indicated that a 2-point increase in SOFA score correlates with an average 10 % increase in mortality [16]. The estimated effect (1.5 points reduction) corresponded to a standardized effect size of 0.5. Assuming usual error rates (α = 0.05; β = 0.20), 64 patients per group were calculated for inclusion. However, due to the non-normal nature of the distribution and the use of nonparametric statistics, the number of patients to be randomized was increased by 10 %. Thus, the total sample size was set to 140 patients (70 per group).

According to the trauma registry of the German Trauma Society (1993 to 2004, n = 20.815), 12 % of multiple-trauma patients with ISS ≥ 16 had a femoral shaft fracture. A level 1 trauma center treats about 50 to 100 severe trauma patients each year. The number of appropriate patients with femur shaft fractures, and thus the feasibility of recruitment, was calculated to be 6 to 12 per year per center.

Participating study centers are listed (see Additional file 1). The study management was provided by the Department of Trauma and Orthopedic Surgery as well as by the Institute for Research in Operative Medicine (IFOM) of the University of Witten-Herdecke at the Campus Cologne-Merheim. The Coordinating Center for Clinical Studies Cologne (KKSK) provided the infrastructure for data management (database MACRO) and internet randomization. Statistical analysis was performed in collaboration with IFOM at the University of Witten-Herdecke. The study was funded by the Deutsche Forschungsgemeinschaft (grant number: RI 929/3-1).

In order to guarantee a high quality of the study and data retrieval, all participating centers were visited on a regular basis (monitoring plans and reports) on site by experienced monitors. Randomly selected patient files were analyzed (100 % source data verification in 15 % of the patients).

The study was approved by the ethics committee of each participating study center (see Additional file 2). The study was conducted according to ICH-GCP (International Conference on Harmonisation for Good Clinical Practice in clinical research), as set out in the European Union Clinical Studies Directive (2001) and associated UK Regulations (2004), which adhere to the principles of the Helsinki Declaration.

Before inclusion, patients were informed about the study. However, at the time of admission, the majority of patients were not able to give consent. In these cases, the patient could be enrolled under waiver of informed consent. This way of enrollment required a “Physician Authorization Form,” where an independent physician and an impartial witness confirmed by signature adherence to all the above-mentioned regulations. This process of enrollment is in accordance with German law and international standards of research. The patient was informed about the study as soon as possible and was asked to sign the applicable informed consent form to continue participation in the study. This consent (or its withdrawal thereof) superseded the authority of any previous authorization for study enrollment. We obtained informed consent from each participant.

Data were analyzed according to the intention-to-treat principle, and therefore, one patient who died before the intervention was started was excluded. Data are presented as mean, median, standard deviation, and range for metric variables. Primary and secondary outcome parameters were compared using nonparametric rank statistics (U-test of Mann and Whitney). Counts were compared with Fisher’s Exact test. A p value < 0.05 was considered statistically significant.

## Results

**Fig. 1:**
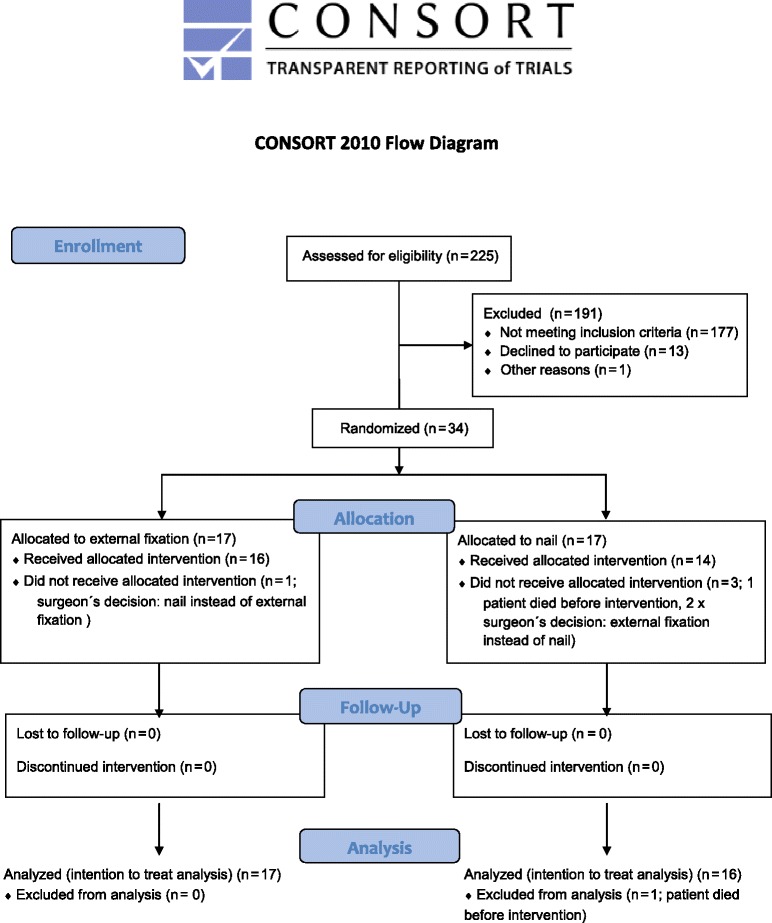
CONSORT 2010 Flow Diagram: Flow diagram for enrollment, allocation, follow-up, and analysis

From June 2007 to December 2009, 249 multiple-trauma patients with femur shaft fracture were screened in 24 of 27 participating trauma centers. 225 patients fulfilled the screening criteria (femoral shaft fracture, ISS ≥ 16, and age ≥ 18 years) (Fig. 1).

**Fig. 2:**
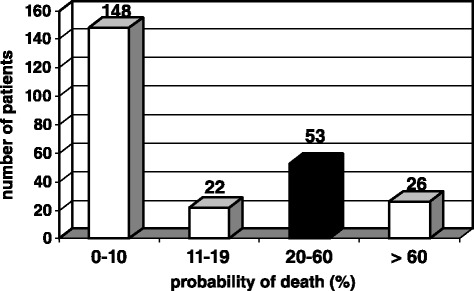
Distribution of probability of death

Of these 225 patients, 53 patients fulfilled the inclusion criteria (femoral shaft fracture, ISS ≥ 16, age ≥ 18 years, and probability of death 20 to 60 %) (Fig. 2). Although the aforementioned inclusion criteria were fulfilled, 19 of these 53 patients were excluded for randomization; five patients met the exclusion criteria, but in more than half of the cases, a subjective decision was made by the responsible surgeon on duty.

**Fig. 3:**
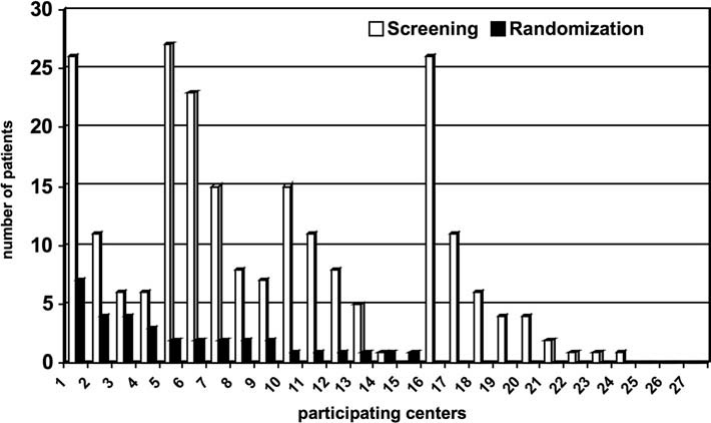
Patient recruitment per trauma center

Finally, 34 patients were included and randomized for the intention-to-treat analysis in 15 of the trauma centers (Fig. 3).

The randomization led to 17 patients per group. The number of patients per center ranged from one to seven. One of the 34 patients died after randomization but before operative treatment, so 33 patients were included in the analysis of postoperative data. All patients were injured by blunt trauma. With respect to the central variables for calculation of the probability of death, both groups were matched by age, ISS, BE, prothrombin time, and GCS on admission. In addition, both groups were matched by physiologic parameters on admission (Table 1). In both groups 94 % of the patients were intubated on admission. Furthermore 12/17 patients were male, and 5/17 patients were female in both groups.

**Table 1:** Comparability of groups on admission

	n = 17 patients with fixateur externe				n = 16 patients with nail			
	mean	median	SD	range	mean	median	SD	range
Age (years)	39.4	40	15.3	18–70	38.9	39	15.3	19–64
ISS (score points)	39.8	41	8.9	18–50	41.4	41	15.7	20–75
Base excess (mmol/L)	−4.9	−5.2	3.7	−9.5–1.7	−6.5	−5.8	4.1	−15– − 0.2
Prothrombin time (%)	66.4	65	23.8	6–100	62.8	60.5	14.2	42–86
GCS (score points)	7.0	6	3.6	3–15	8.5	8	3.2	3–14
Calculated probability of death (%)	31	25	13	20–54	30	26	12	20–59
Systolic blood pressure (mm Hg)	112	108	29	70–160	107	115	41	60–180
Heart rate (beats/min)	96	100	25	40–130	109	107	24	78–145
Respiratory rate (per min)	14.4	12	6.5	5–30	12.3	12	5.5	0–20
SpO_2_	85.9	92	20.0	14–100	87.3	95	24.8	0–100

*SD* standard deviation, *ISS* injury severity score, *GCS* Glasgow Coma Scale

In 3/33 cases (9 %), the surgeon decided to deviate to the alternative treatment modality after randomization. Two patients were randomized to intramedullary nailing but were treated with external fixation. In one of these cases, the surgeon explained his deviation from protocol by the patient’s highly unstable circulatory parameters and, in the other case, by the fact that the patient suffered from traumatic head injury with the necessity for head elevation because of strong nasal bleeding. In a third patient, the surgeon felt that the patient’s circulation was too stable to justify external fixation and thus performed femoral nailing.

The primary endpoint (maximal SOFA-score) was increased by 0.9 points in the nail-group, but this difference was not significant. Thus, the expected difference of at least 1.5 score points between the groups (according to the study protocol) was not reached. Table 2 compares the primary endpoint and the most important secondary endpoints.

**Table 2:** Primary and secondary endpoints

	n = 17 patients with fixateur externe				n = 16 patients with nail				p value
	mean	median	SD	range	mean	median	SD	range	
Maximal SOFA score	8.7	9	3.8	1-20	9.6	9.5	5.1	2-20	0.510
Cumulative SOFA score	112.4	84	118.8	2-517	113.8	51	166.6	4-544	0.254
Transfusion requirements during surgery (packed red blood cells)	4.7	2	4.8	0-16	6.6	4	6.1	0-17	0.350
ICU length of stay (days)	21.8	20	13.9	3-54	12.38	9.5	9.9	2-40	0.037
Days of ventilation	15.0	15	9.6	0-28	8.6	6	7.9	1-28	0.049
In-hospital length of stay (days)	32.3	28	20.2	6-84	30.2	26.5	18.2	3-77	1.0

*SD*, standard deviation, *SOFA* sepsis-related organ failure assessment, *ICU* intensive care unit

Transfusion requirements during the operation were comparable between both groups (Table 2). Whereas patients with external fixation required a significantly longer ventilation period (p = 0.049) and stayed in the intensive care unit significantly longer (more than 1 week on average; p = 0.037), the in-hospital length of stay was balanced again between both groups (n.s.) because the patients in the nail-group stayed in the normal ward longer.

According to the intention-to-treat analysis, the rates of SIRS (15 in fixateur externe versus 14 in nail group), sepsis (four in the external fixation versus two in the nail group) and ARDS (none in the external fixation versus two in nail group) were comparable between both groups (n.s.).

Overall, three patients (9 %) died, one in the external fixation group and two in the nail group. However, the two nonsurvivors in the nail group were those in whom the treating surgeon decided to deviate from the randomized procedure. Thus, according to the “as-treated” principle all deaths occurred in the external fixation group.

**Fig. 4:**
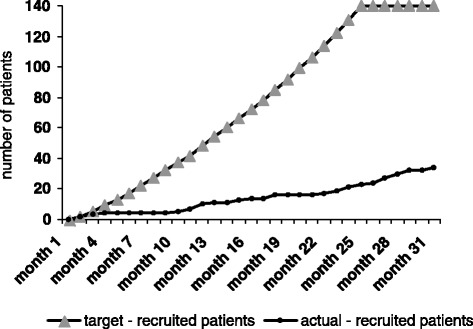
Patient recruitment over time

Unfortunately, the study had to be terminated prematurely before reaching the proposed sample size because not enough patients could be recruited for randomization in a timely fashion, and funding was then stopped by the Deutsche Forschungsgemeinschaft. During the study, we recognized that the target patient population was smaller than anticipated. In addition, obtaining the testing protocol-required laboratory parameters, obtaining third-party consent, and performing randomization turned out to be difficult to perform during the short time interval between hospital admission and surgery. Thus, while the ratio of included patients to screened patients were roughly equivalent to the calculations of the study protocol (1:10), the total number of screened/enrolled patients remained well behind the underlying prognosis (Fig. 4).

## Discussion

Today, a trend toward the concept of “damage control orthopedics” exists in the management of multiple-trauma patients with long bone fractures. Nevertheless, evidence from the current literature is insufficient, and a generalized management strategy is missing. The present study was obviously too small in the sample size to detect a difference in the maximum SOFA score. Therefore, the most interesting findings are the difference in length of ICU stay and the three fatalities. Many surgeons believe that early total care allows for a quicker recovery, but some argue that this is at the expense of a slightly higher mortality rate at the initial surgery. The present results partly refute these fears; however, the number of deaths was small, and some borderline patients may have been excluded from the trial before inclusion.

While this study was not the first to evaluate damage control orthopedic surgery of the femur shaft fractures in multiple-trauma patients in a randomized controlled design, it was the first study to concentrate only on a “borderline” population with an extremely high severity of injury and physiologic derangement. In 2003, Pape et al. [19] presented the results of their randomized controlled study. They investigated the impact of intramedullary instrumentation versus damage control for femoral fractures on immunoinflammatory parameters and complications [19–21]. However, in contrast to the present study, where a risk adaption (probability of death of 20 to 60 %) was performed, they excluded multiple trauma patients with severe brain and thoracic injuries (AIS > 3), as well as patients in unstable or critical condition. In summary, the included patients were injured less severely and only the subpopulation of patients in a borderline condition profited from the damage control approach. Furthermore, Pape et al. did not define criteria that must be fulfilled for performing the secondary definitive procedure. Therefore these two studies are not comparable. The present study is rather a further development. A significant effect could only be expected in a subgroup of medium probability of death (20 to 60 %), especially related to the maximum SOFA-score, as the type of procedure chosen in patient groups of very high or very low mortality will most likely have only a minimal effect on this endpoint.

Although mortality would have been the most appropriate endpoint, a study with mortality as the main endpoint would need approximately 1,300 patients per arm. In addition to the fact that such a study is almost impossible to perform for practical reasons the focus on mortality, however, does not cover all aspects of the planned intervention since the damage control approach primarily tries to limit the sequelae of the “second hit” by surgical intervention. This is reflected by the measurement of organ failure as a surrogate endpoint by appointing maximum values for patients who died. In addition, because the most important factors that determine prognosis in multiple-trauma patients are considered, a comparison of this heterogenic patient collective is possible.

In the present trial, some surgeons were either unwilling to include all eligible patients or decided to deviate from the allocated treatment method. This shows that personal beliefs and pathophysiologic reasoning are strongly interfering with the choice of management strategy. Due to the small sample size of the present study, an examination of whether specific subgroups truly fare better when receiving a femoral nail or an external fixation was not possible. Future studies should therefore pay attention to these specific subgroups, for example, patients with higher injury severity or specific injuries to the head, thorax, or pelvis.

## Conclusion

In conclusion, the results of this randomized study reflect the ambivalence in the literature. In correspondence to the systematic review [4], we could not find advantages of the damage control concept in the treatment of femoral shaft fractures in the care of multiple trauma patients. Unfortunately, our results are not statistically significant due to the small number of included patients. Thus, the necessity for scientific evaluation of this clinically relevant question remains.

## Additional files

Additional file 1:
**Participating centers.** (DOC 60 kb) Additional file 2:
**Ethical bodies.** (DOC 33 kb)

## Abbreviations

AIS: Abbreviated Injury Scale
ARDS: Adult Respiratory Distress Syndrome
BE: base excess
CNS: central nervous system
CRF: case report form
DCO: damage control orthopedics
DSMB: Data Safety Monitoring Board
ETC: early total care
GCS: Glasgow Coma Score
GCP: good clinical practice
ICU: intensive care unit
ISS: Injury Severity Score
KKSK: Coordinating Center for Clinical Studies Cologne
MODS: Multi-organ Dysfunction Syndrome
PI: primary investigator (Principle Coordinating Investigator)
SDV: source data verification
SIRS: Systemic Inflammatory Response Syndrome
SOFA: Sepsis-related Organ Failure Assessment
TISS: Therapeutic Intervention Scoring System
