# Supplementary material for: Randomized, controlled, two-arm, interventional, multicenter study on risk-adapted damage control orthopedic surgery of femur shaft fractures in multiple-trauma patients
Source: Trials. 2016 Jan 25;17:47. doi: 10.1186/s13063-016-1162-2 (PMC4727266; doi:10.1186/s13063-016-1162-2)
Supplement: Additional file 1: — Participating centers. (DOC 60 kb) [file 13063_2016_1162_MOESM1_ESM.doc]

**Participating trauma centers (in alphabetical order):**

Department of Trauma and Reconstructive Surgery, **Unfallkrankenhaus Berlin (UKB)**

Center for Musculosceletal Surgery, Department of Trauma and Reconstructive Surgery, **University of Berlin Charite Campus Virchow Clinic**

Department of Trauma, Hand and Reconstructive Surgery, **Helios Clinic Berlin-Buch**

**Department of Surgery -** Trauma and Reconstructive Surgery **Vivantes-Clinic Berlin**

Department of Orthopedic and Trauma Surgery, **University of Bonn**

Department of Trauma Surgery, Orthopedics and Sporttraumatology, **University of Witten-Herdecke at the Campus Cologne-Merheim**

Department of Trauma, Hand and Reconstructive Surgery, **University of Cologne**

Department of Trauma and Hand Surgery, **University of Düsseldorf**

Department of Trauma Surgery, **University of Essen**

Department of Trauma, Hand and Reconstructive Surgery, **Johann Wolfgang Goethe University Frankfurt**

Center for Trauma Surgery**, Berufsgenossenschaftliche Unfallklinik Frankfurt**

Department of Trauma Surgery **University of Halle**

Department of Trauma, Hand and Reconstructive Surgery, **University of Hamburg**

Department of Trauma Surgery **Medical University of Hannover**

Department of Trauma Surgery, **Surgical University Hospital Heidelberg**

Department of Trauma, Hand and Reconstructive Surgery **University of Saarland (Homburg)**

Department of Trauma Surgery, **Clinic Ingolstadt**

Department of Trauma Surgery, **University Hospitals Schleswig-Holstein, Campus Kiel**

Department of Trauma and Reconstructive Surgery, **University of Leipzig**

Department of Trauma Surgery,  **Berufsgenossenschaftliche Unfallklinik Ludwigshafen**

Department of Trauma Surgery, **University Hospitals Schleswig-Holstein, Campus Lübeck**

Department of Trauma Surgery, **St. Marien-Hospital Lünen**

Department of Trauma, Reconstructive and Hand Surgery, **University of Marburg/Gießen**

Department of Trauma Surgery, **University of Munich, Campus Innenstadt**

Department of Trauma Surgery, **University of Munich, Campus Großhadern**

Department of Trauma Surgery, **University of Regensburg**

Department of Trauma and Reconstructive Surgery, **University of Rostock**
