# Supplementary material for: Randomized, controlled, two-arm, interventional, multicenter study on risk-adapted damage control orthopedic surgery of femur shaft fractures in multiple-trauma patients
Source: Trials. 2016 Jan 25;17:47. doi: 10.1186/s13063-016-1162-2 (PMC4727266; doi:10.1186/s13063-016-1162-2)
Supplement: Additional file 2: — Ethical bodies. (DOC 33 kb) [file 13063_2016_1162_MOESM2_ESM.doc]

**Ethical bodies (in alphabetical order)**

Ethik-Kommission **Unfallkrankenhaus Berlin**

Ethikkommission der Charite – Universitätsmedizin Berlin

Ethikkommission **der Helios Klinik Berlin-Buch**

Ethikkommission der Vivantes-Klinik Berlin

Ethikkommission Universitätsklinikum Bonn

Ethikkommission Universität Düsseldorf

Ethik-Kommission Universität **Duisburg-Essen**

Ethikkommission **Johann Wolfgang Goethe** Universität **Frankfurt**

Ethikkommission der **Berufsgenossenschaftlichen Unfallklinik Frankfurt**

Ethik-Kommission Universität **Halle**

Ethikkommission Universität Hamburg

Ethikkommission **Medizinische Hochschule Hannover**

Ethikkommission Universität Heidelberg

Zentrale Ethikkommission der Universität des Saarlandes in Homburg

Ethikkommission Klinikum **Ingolstadt**

Ethikkommission Universitätsklinikum **Schleswig-Holstein, Campus Kiel**

Ethikkommission – Medizinische Fakultät – Universität zu Köln

Ethik-Kommission der U**niversität Witten-Herdecke, Campus Köln**

Ethik-Kommission Universität Leipzig

Ethikkommission der Berufsgenossenschaftlichen Unfallklinik Ludwigshafen

Ethikkommission Universitätsklinikum **Schleswig-Holstein, Campus Lübeck**

Ethikkommission des **St. Marien-Hospital Lünen**

Ethikkommission Universität Marburg/Gießen

Ethikkommission Ludwig-Maximilians Universität München, Campus Innenstadt

Ethikkommission Ludwig-Maximilians Universität München, Campus Großhadern

Ethikkommission an der Universität Regensburg

Ethikkommission Universität Rostock
